# Effect of proteins isolated from Brazilian snakes on enterovirus A71 replication cycle: An approach against hand, foot and mouth disease

**DOI:** 10.1016/j.ijbiomac.2023.124519

**Published:** 2023-04-19

**Authors:** Jacqueline Farinha Shimizu, Shiraz Feferbaum-Leite, Igor Andrade Santos, Daniel Oliveira Silva Martins, Natalie J. Kingston, Mona Shegdar, Carsten Zothner, Suely Vilela Sampaio, Mark Harris, Nicola J. Stonehouse, Ana Carolina Gomes Jardim

**Affiliations:** a Laboratory of Antiviral Research, Institute of Biomedical Science — ICBIM, Federal University of Uberlândia — UFU, Uberlândia, MG, Brazil; b Institute of Biosciences, Language and Exact Science — IBILCE, São Paulo State University — UNESP, São José do Rio Preto, SP, Brazil; c Brazilian Biosciences National Laboratory (LNBio), Brazilian Centre for Research in Energy and Materials (CNPEM), Campinas, SP 13083-100, Brazil; d School of Molecular and Cellular Biology, Faculty of Biological Sciences and Astbury Centre for Structural Molecular Biology, University of Leeds, Leeds LS2 9JT, United Kingdom; e Department of Clinical Analyses, Toxicology and Food Sciences, School of Pharmaceutical Sciences of Ribeirão Preto, University of São Paulo — USP, SP, Brazil

**Keywords:** Enterovirus A71, Antiviral, Brazilian snakes, Toxins, EVA71

## Abstract

Enterovirus A71 (EVA71) belongs to the *Picornaviridae* family and is the main etiological agent of hand, foot, and mouth disease (HFMD). There is no approved antiviral against EVA71, and therefore the search for novel anti-EVA71 therapeutics is essential. In this context, the antiviral activity of proteins isolated from snake venoms has been reported against a range of viruses. Here, the proteins CM10 and CM14 isolated from *Bothrops moojeni*, and Crotamin and PLA2_CB_ isolated from *Crotalus durissus terrificus* were investigated for their antiviral activity against EVA71 infection. CM14 and Crotamin possessed a selective index (SI) of 170.8 and 120.4, respectively, while CM10 and PLA2_CB_ had an SI of 67.4 and 12.5, respectively. CM14 inhibited all steps of viral replication (protective effect: 76 %; virucidal: 99 %; and post-entry: 99 %). Similarly, Crotamin inhibited up to 99 % of three steps. In contrast, CM10 and PLA2_CB_ impaired one or two steps of EVA71 replication, respectively. Further dose-response assays using increasing titres of EVA71 were performed and CM14 and Crotamin retained functionality with high concentrations of EVA71 (up to 1000 TCID_50_). These data demonstrate that proteins isolated from snake venom are potent inhibitors of EVA71 and could be used as scaffolds for future development of novel antivirals.

## 1 Introduction

Enterovirus A71 (EVA71) is one of the major causes of hand, foot, and mouth disease (HFMD), characterized by fever and rash with blisters on the hands, feet, buttocks, and mouth [1]. EVA71 infection may also be associated with more severe cases of encephalitis, aseptic meningitis, and myocarditis, which could result in long-term sequelae and high mortality rates, especially in children [2–5]. EVA71 belongs to the *Enterovirus* genus from the *Picornaviridae* [6]. It is a non-enveloped virus, with an icosahedral capsid and a single-stranded positive RNA genome [7]. The viral RNA contains one open reading frame (ORF) that encodes a polyprotein cleaved into four structural proteins (VP1, VP2, VP3, and VP4) and seven non-structural proteins (P2-2A, 2B, 2C, P3-3A, 3B, 3C, and 3D) [8–11].

Transmission occurs mainly via fecal-oral or oropharyngeal secretions, being highly contagious. In addition, it can also contaminate surfaces and promote transmission by fomites [12]. Interestingly, the incidence increases at the warmest times of the year [13]. EVA71 replicates in gastrointestinal and respiratory epithelium cells, however, the viremia can result in infection of neurons and cardiomyocytes [14]. An infected individual can be asymptomatic, and in most cases the infection is self-limiting, however, symptomatic cases can progress to HFMD. Thus, the infection may reach the central nervous system and cause dysregulation of the autonomic nervous system, resulting in cardiopulmonary failure in more severe cases.

Among the causes of HFMD, EVA71 has become the most relevant because of its neurotropism, which can trigger diseases associated with the neurological system, such as aseptic meningitis, encephalitis, poliomyelitis syndrome, and acute flaccid paralysis [15,16]. In severe cases, EVA71 can affect the brainstem, causing sequelae and even death [17]. This virus has been responsible for outbreaks associated with neurological complications in children [18,19]. In recent years, epidemics in Southeast Asian countries generated concern about the potential of EVA71 dissemination in highly populated regions. In 2012, China registered >2 million cases and 567 deaths related to HFMD [20]. Since the emergence of the coronavirus disease in 2019 (COVID-19), the application of non-pharmacological control measures (social distancing, masks, and hand sanitization) decreased the incidence of HFMD in 2020 [21]. However, new outbreaks in children were reported in 2022 due to the re-establishment of common activities [22].

Currently, there is no approved antiviral therapy against HFMD [23,24], and only one vaccine approved in China against HFMD subgenotype C4 [25–27]. In this context, the search for therapeutics against EVA71 is essential and could provide substantial health benefits. Proteins isolated from animal venoms have shown to be a fruitful source of molecules with several biological activities, such as anti-inflammatory, anti-cancer, and cardiovascular activity [28–30]. Among those, proteins isolated from Brazilian snake venoms, such as *Bothrops moojeni* and *Crotalus durissus terrificus* [31] have shown antiviral activity against hepatitis C virus (HCV) [32], dengue virus (DENV) and yellow fever virus (YFV) [33], measles virus [34], Chikungunya virus (CHIKV) [35], and ZIKV [36]. Therefore, the biological properties presented by the constituents of snake venoms represent promising sources for the identification and development of drugs against emergent viruses.

In this study, the antiviral activity of proteins CM10, CM14, Crotamin, and PLA2_CB_ isolated from Brazilian venom snakes *B. moojeni* and *C. durissus terrificus* were investigated against the EVA71 replication cycle.

## 2 Material and methods

### 2.1 Venoms and isolated proteins

The crude venom of *Bothrops moojeni* was purchased from serpentarium “Serpentário Proteínas Bioativas Ltda” of Batatais/SP, registered at the Ministry of the Environment, n° 471301. For *Crotalus durissus terrificus*, the crude venom was purchased from serpentarium “Centro de Extração de Toxinas Animais”, registered at the Ministry of the Environment, n° 3002678. Isolation and purification of the proteins Crotamim and PLA2_CB_ were carried out at the Laboratory of Toxinology of the School of Pharmaceutical Sciences of Ribeirão Preto, University of São Paulo (IBAMA authorization: 1/35/1998/000846-1), as previously described [37], using the Itzhaki and Gill method [38]. CM14 and CM10 were isolated from the crude venom of *Bothrops moojeni* by ion-exchange chromatography on CM-Sepharose (Pharmacia) followed by reverse phase chromatography as previously described [39,40]. The evaluation of the purification efficiency was monitored by 12 % SDS-PAGE and Coomassie blue R250 staining [41]. Lyophilized proteins were dissolved in PBS (Phosphate-Buffered Saline), filtered, aliquoted, and stored at −80 °C. Stocks were diluted in DMEM immediately prior to the experiments. PBS was used as untreated control. The source of each protein is shown in Table 1.

### 2.2 Cell culture

Vero E6 cells (kidney tissue derived from a normal adult African green monkey, ATCC E6) were grown in minimal Dulbecco’s Modified Eagle medium (DMEM; Sigma-Aldrich, USA) supplemented with 100 U/mL penicillin (Gibco Life Technologies, USA), 100 U/mL streptomycin (Gibco Life Technologies, USA), 1 % HEPES (Gibco Life Technologies, USA), and 10 % fetal bovine serum (FBS; Gibco Life Technologies, USA) at 37 °C in a humidified incubator with 5 % CO_2_.

### 2.3 Virus rescue and titration

An EVA71 reverse genetics system was generated from the strain MS/7423/87 as previously described [42]. The plasmids were linearized with *Xho*I (ThermoFisher Scientific) and purified using Wizard® DNA Clean-Up System (Promega). The linearized DNA was transcribed using the HiScribe™ T7 High Yield RNA Synthesis Kit (NEB). The infectious RNA was electroporated into Vero cells (1 × 10^6^ cells) at 250 μF and 450 V. After 48 h, the supernatant was harvested and titrated as follows: Vero cells were seeded in 96 well plates at 1 × 10^4^ cells/well 24 h prior to infection with EVA71 cell culture supernatants diluted in a 10-fold serial dilution in DMEM. After incubation for 48 h cells were fixed with 4 % paraformaldehyde (PFA) and stained with crystal violet 0.5 % for 30 min. Titres were determined using the 50 % tissue culture infectious dose (TCID_50_) method and calculated by the Spearman & Kärber algorithm as described by Killington and Hierholzer [43]. All work was performed at biosafety level 2 under the authorization number SEI: 01245.006267/2022-14 CBQ: 163/02 from the CTNBio — National Technical Commission for Biosecurity from Brazil.

### 2.4 Cell viability assays

Cell viability was measured by the MTT [3-(4,5-dimethylthiazol-2-yl)-2,5-diphenyl tetrazolium bromide] (Sigma-Aldrich) method [35,44,45]. Vero cells were seeded in a 96 well plate at 1 × 10^4^ cells/well and incubated overnight at 37 °C in a humidified 5 % CO_2_ incubator. Medium-containing compounds at 50, 25, 5, and 1 μg/mL was added to the cell culture and incubated for 72 h. The supernatant was removed and DMEM containing MTT at 1 mg/mL was added to each well, incubated for 30 min at 37 °C in a humidified 5 % CO_2_ incubator, and replaced with 100 μL of DMSO to solubilize the formazan crystals. PBS was used as untreated control. The absorbance was measured at 560 nm on Glomax Explorer (Promega). The cell viability was determined in percentage by the average of treated wells × 100/average of control wells. To determine the cytotoxic concentration of 50 % (CC_50_), the proteins were diluted in 2 % DMEM in two-fold serial dilution series from 1.2 to 150 μg/mL for CM14, CM10, and Crotamin, and from 0.4 to 50 μg/mL for PLA2_CB_, then added to the cells for cell viability assays, as described above.

### 2.5 Antiviral assays

To evaluate the anti-EVA71 activity of proteins isolated from snake venom, a suspension of Vero cells was plated in 96 well microplates at a concentration of 1 × 10^4^ cells/well and incubated at 37 °C and 5 % CO_2_ for 24 h. The compounds were diluted in a medium containing EVA71 at 100 TCID_50_ at specific concentrations and then added to the cells for 72 h. Then, the supernatant was removed and 100 μL of a solution containing MTT (Sigma-Aldrich) at 1 mg/mL of culture medium was added to the cells, which were incubated at 37 °C for 30 min. The MTT solution was removed, replaced by the same volume of DMSO, and the absorbance was measured at 560 nm on GloMax plate reader (Promega). PBS was used as an untreated control. The inhibition of EVA71 replication in cells by each protein was calculated using the following formula [(ODt) EVA71 − (ODc)EVA71] / [(Odc)cell − (Odc)EVA71] × 100 (%), where (ODt)EVA71 is the measured optical density of treatment on EVA71 infected cells; (ODc)EVA71 is the optical density measured for untreated control EVA71 infected cells; and (Odc)cell is the measured optical density of uninfected control cells [46]. Antiviral activity was presented as % inhibition of EVA71 replication. To determine the effective concentration of 50 % (EC_50_), CM14, CM10, Crotamin, and PLA-2C_B_ were diluted in a culture medium containing EVA71 at 100 TCID_50_, in a two-fold serial dilution with concentrations ranging from 1.2 to 150 μg/mL for CM14, CM10, and Crotamin, and from 0.4 to 50 μg/mL for PLA2_CB_, and the antiviral assay was performed as described above.

### 2.6 Time of addition assays

The activity of the proteins isolated from snake venoms was evaluated at different stages of the EVA71 replicative cycle. To evaluate whether compounds presented a protective effect against EVA71 infection, Vero cells were treated with each compound for 1 h at 37 °C in a humidified 5 % CO_2_ incubator prior to the infection. After incubation, cells were washed extensively to remove compounds and were infected with EVA71 for 1 h. The infectious supernatant was removed, additional washes were performed for virus removal and fresh media was added.

For the virucidal assay, the infectious supernatant was incubated with each protein for 1 h at 37 °C, prior to use for infection of Vero cells. Virus and protein were incubated with cells for 1 h at 37 °C. The inoculum was removed; cells were washed three times with PBS to completely remove the virus and proteins and replaced with fresh media.

To investigate the antiviral activity on EVA71 replication, infectious supernatant was added to Vero cells for 1 h, washed with PBS to remove non-endocytosed virus particles, and replaced by compound-containing media.

For all antiviral assays, the supernatant was collected 72 h post-infection (h.p.i.) and used to infect Vero cells in 96 well plates. Cells were washed with PBS after 1 h and incubated with fresh media for 72 h. The titres were determined by the TCID_50_ method as described above.

### 2.7 Western Blot

Cells were lysed in Passive Lysis Buffer (PLB, Promega) with added protease inhibitors (Sigma-Aldrich). The protein concentration of samples was calculated using Pierce BCA Protein Assay Kit (Thermofisher). Ten micrograms of protein were resolved by SDS-PAGE and transferred to a PVDF membrane. Membranes were blocked in 10 % (w/v) dried skimmed milk powder in Tris-buffered saline with 0.1 % Tween-20 (TBS-T). Membranes were probed with mouse anti-enterovirus VP0 (anti-VP0) monoclonal antibody at a 1:2000 dilution (mAb979, Millipore), secondary anti-mouse horseradish peroxidase-conjugated antibody (Sigma) and ECL substrate (Thermo scientific).

### 2.8 TCID_50_ challenge in vitro assay

To investigate the inhibitory potential of the proteins in the presence of high amounts of EVA71, Vero cells were infected with increasing concentrations of virus from 1 TCID_50_ to 10,000 TCID_50_ in the presence of the proteins at the highest non-cytotoxicconcentration (CM10, CM14, and Crotamin at 50 μg/mL, and PLA2_CB_ at 5 μg/mL). Then, the supernatant was removed 48 h.p.i. and 100 μL of a solution containing MTT (Sigma-Aldrich) at 1 mg/mL of culture medium was added to the cells, which were incubated at 37 °C for 30 min. Antiviral activity was determined as described above (antiviral assays section) [46] and was presented as % inhibition of EVA71 replication. PBS was used as an untreated control.

### 2.9 Statistical analysis

All assays were performed a minimum of three times in order to confirm the reproducibility of the results. Differences between means of readings were compared using analysis of variance (One-way or Two-way ANOVA) and Student *t*-test. *p* values of <0.05 (indicated by asterisks) were considered statistically significant. For the establishment of EC_50_ and CC_50_ values, the data were transformed into Log(X), where X is the concentration, and submitted into a non-linear regression with four parameters in variable slope. All analyses were performed using GraphPad Prism 8.

## 3 Results

### 3.1 Purification of the isolated proteins

**Fig. 1:**
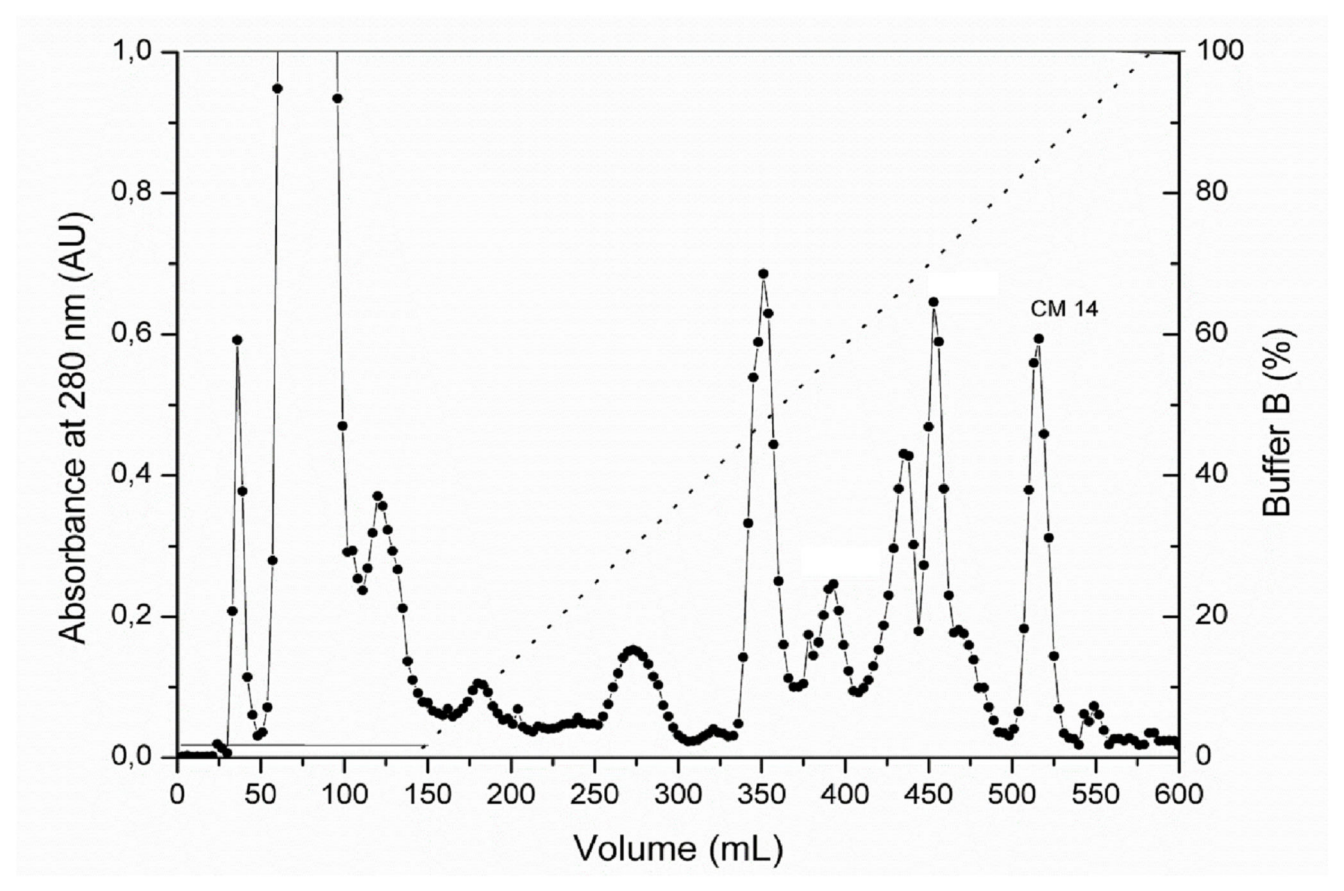
Isolation of CM14 from *Bothrops moojeni* snake venom. Chromatographic profile of *B. moojeni* crude venom (200 mg) on a CM Sepharose column, equilibrated and eluted with 50 mM ammonium bicarbonate buffer, pH 7.8, followed by a linear gradient of up to 500 mM (buffer B). Fractions were collected a flow rate of 20 mL/h and at room temperature.

**Fig. 2:**
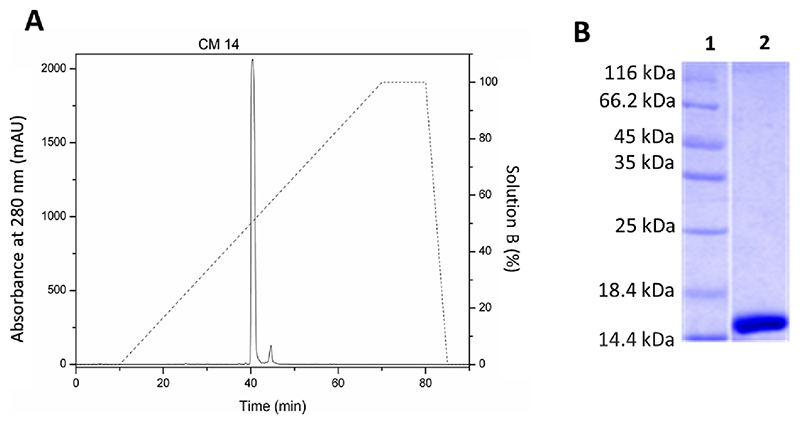
Chromatographic profile of CM14. CM14 was separated on a C18 reversed-phase column equilibrated and eluted with 0.1 % trifluoroacetic acid (TFA), and 70 % acetonitrile and 0.1 % TFA (solution B). Protein elution was achieved at flow rate of 1 mL/min with a linear concentration gradient of solution B (A). SDS-PAGE of the purified CM14 (2) under denaturing and reducing conditions. (1) Molecular weight standard: beta-galactosidase (116 kDa), bovine serum albumin (66.2 kDa), ovalbumin (45.0 kDa), lactate dehydrogenase (35.0 kDa), REase Bsp98I (25.0 kDa), beta-lactoglobulin (18.4 kDa), lysozyme (14.4 kDa) (B).

The process of isolation and characterization of PLA2_CB_ and Crotamin from *Crotalus durissus terrificus* venom, used in this study, was previously described by Muller and colleagues [33,37]. The protein CM10 was isolated from *Bothrops moojeni*, and its purification and characterization were published by Amorin and coworkers [40]. The protein CM14 was purified from *Bothrops moojeni* venom using the same protocol previously described for CM10 [40], which represents a two-step chromatographic process. Briefly, the first step was performed on a CM sepharose cation-exchange column (Fig. 1), in which the peak corresponding to CM14 is indicated (Fig. 1). Then, the fraction of CM14 was subjected to C18 reversed-phase chromatography (Fig. 2A) resulting in a major fraction, revealing a single band (Fig. 2B). Through the biochemical characterization using SDS-PAGE, pI (data not shown) and N-terminal amino acid sequencing (data not shown) CM14 was identified as myotoxin II, according to [47].

### 3.2 CM10, CM14, Crotamin and PLA2_CB_ strongly impair EVA71 with high selectivity index

**Fig. 3:**
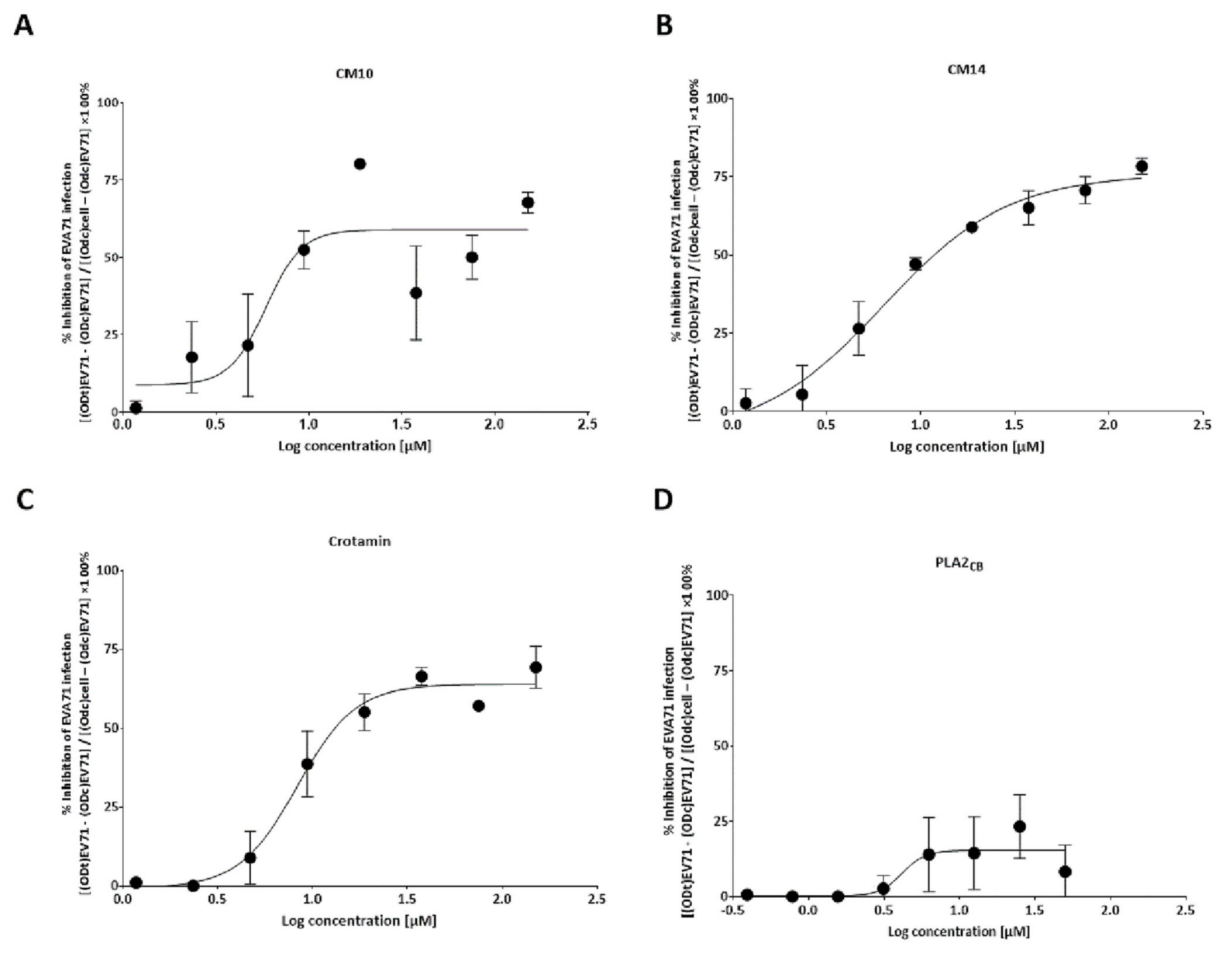
EC_50_ of each snake venom protein. CM10 (A), CM14 (B), Crotamin (C), and PLA2_CB_ (D). Vero cells were treated with CM10, CM14, and Crotamin, with concentrations ranging from 1.2 to 150 μg/mL, and PLA2_CB_ from 0.4 to 50 μg/mL, in the presence of EV-A71 (100 TCID_50_) and added to the cells for 72 h. PBS was used as untreated control. After 72 h, supernatant was removed and MTT solution was added to cells. The MTT solution was removed, replaced by the same volume of DMSO, and read at a wavelength of 490 nm on the GloMax plate reader (PROMEGA). The results were then transformed into percentages, and the inhibition of EV-A71 replication in cells by each protein was calculated using the following formula [(ODt)EVA71 − (ODc)EVA71] / [(Odc)cell − (Odc)EVA71] × 100 (%), where (ODt)EVA71 is the measured optical density of treatment on EV-A71 infected cells; (ODc)EVA71 is the optical density measured for untreated control EV-A71 infected cells; and (Odc)cell is the measured optical density of uninfected control cells [46]. Antiviral activity was presented as % inhibition of EV-A71 replication. Results are shown as a non-linear regression analysis, indicating the percentages of the inhibition of viral replication by each protein. Mean values ± SD of two independent experiments each measured in triplicate are shown. The comparison of each curve was performed employing the F test (confidence level of 95 %), resulting in a *p* < 0.0001.

In order to assess the inhibitory potential of these proteins on the EVA71 replicative cycle, a dose-response assay was performed by treating cells with a two-fold serial dilution ranging from 1.2 to 150 μg/mL for the compounds CM10, CM14, and Crotamin, and from 0.4 to 50 μg/mL for PLA2_CB_, in the presence or absence of 100 TCID_50_ of EVA71. The EC_50_, CC_50_ and calculated selective index (SI) for the proteins are shown in Fig. 3A−D and Table 1. From this data and based on previously performed cytotoxicity assays (Supplementary Fig. 1), non-toxic concentrations of each protein were selected for further antiviral assays (Table 2): CM10, CM14, and Crotamin were used at 50 μg/mL and PLA2_CB_ at 5 μg/mL.

### 3.3 CM10, CM14, Crotamin and PLA2CB inhibit multiple stages of the EVA71 life cycle

**Fig. 4:**
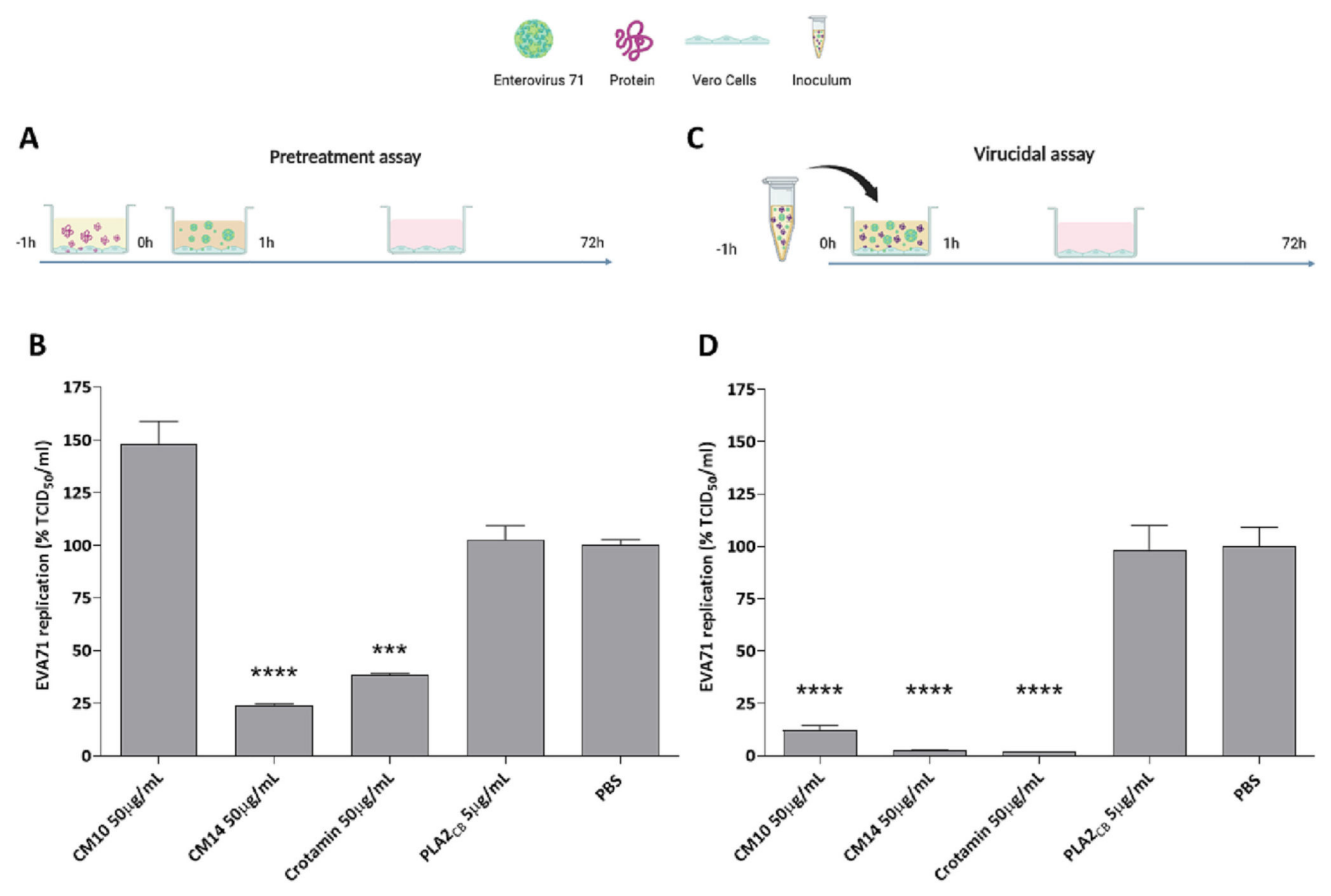
Protective and virucidal effects of proteins against EVA71 infection. EVA71 at MOI of 0.1 and the proteins CM10, CM14, and Crotamin at 50 μg/mL, and PLA2_CB_ at 5 μg/mL were added in different times to the cells and virus replication was titrated by TCID_50_ method. For the pre-treatment assay, Vero cells were treated with each protein for 1 h, washed with PBS, and infected with EVA71 for 1 h. Then, cells were washed and replaced with fresh media and incubated for 72 h (A), and supernatant was collected, titrated, and normalized with PBS control (B). For virucidal assay, EVA71 were incubated with proteins for 1 h prior to the infection on Vero cells. Cells were added of the mixture for 1 h, washed and fresh media was added, cells were incubated for 72 h (C), supernatant was collected, titrated, and normalized with PBS control (D). PBS was used in the untreated infected control. Statistically significant differences between each compound and PBS control are shown. Significant differences from the control are denoted by *** (*p* ≤ 0.001) and **** (*p* ≤ 0.0001).

Time of drug addition assays were employed to identify the activity of each protein in distinct steps of the EVA71 replication cycle. Firstly, Vero cells were pre-treated with each protein for 1 h, washed with PBS to remove the compounds, and followed by infection with EVA71 for 1 h. The supernatant was replaced by fresh media after further PBS washes, and cells were incubated for 72 h (Fig. 4A). The results demonstrated that CM14 and Crotamin reduced virus titres by 76 % and 62 % (*p* < 0.001), respectively (Fig. 4B). In contrast, pre-treatment with CM10 and PLA2_CB_ did not protect cells against EVA71 infection (Fig. 4B).

To evaluate the virucidal activity, an inoculum containing infectious supernatant and each protein was incubated for 1 h, then added to Vero cells for further 1 h. The inoculum was removed, cells were washed with PBS, and replaced with fresh media for 72 h (Fig. 4C). Crotamin and CM14 inhibited 99 % of virus infectivity (*p* < 0.001), while CM10 significantly reduced virus infectivity to 88 % (Fig. 4D). However, PLA2_CB_ did not inhibit virus infectivity (Fig. 4D).

**Fig. 5:**
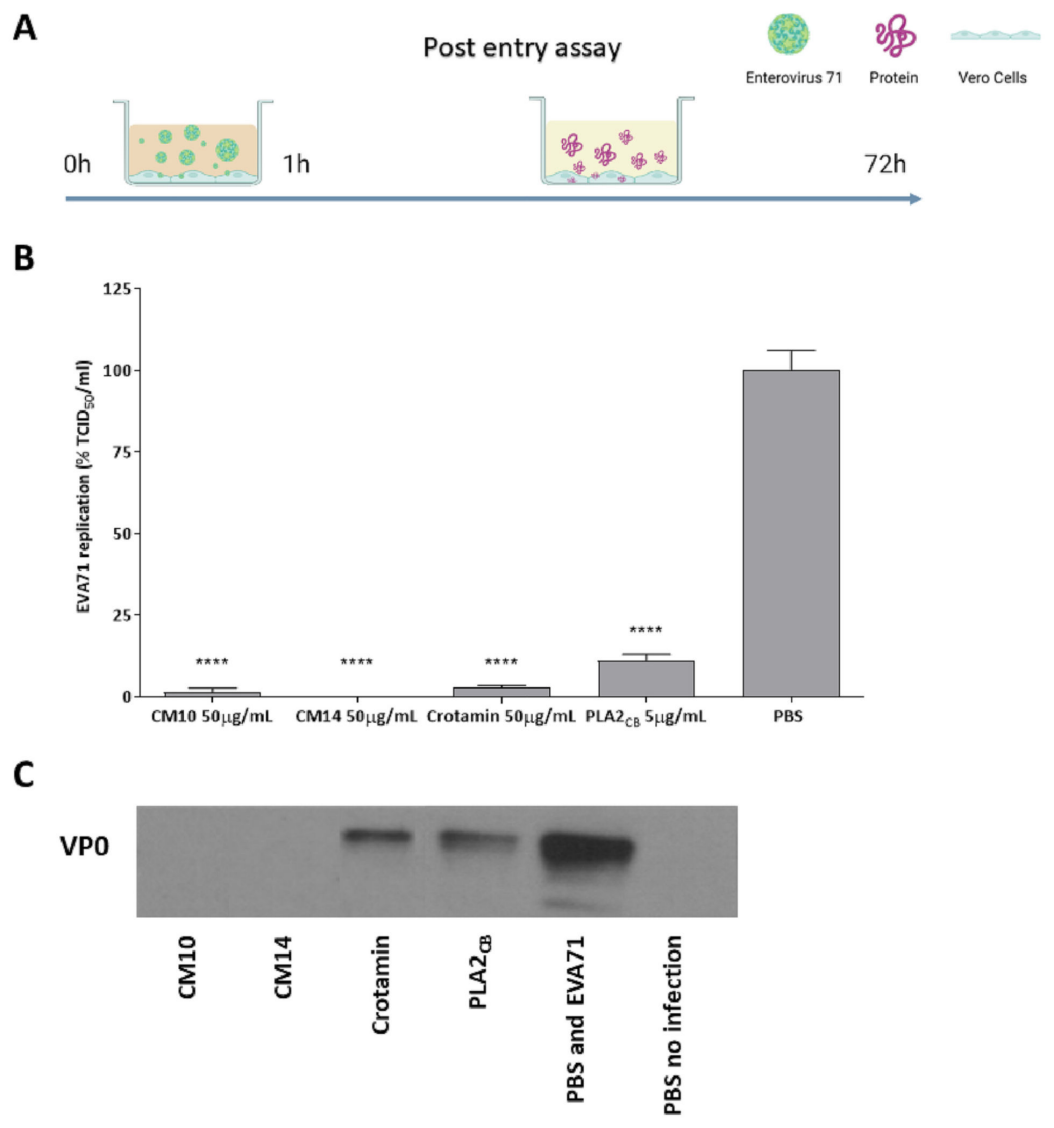
Effect of CM10, CM14, Crotamin, and PLA2_CB_ on EVA71 replication. Vero cells were infected with EVA71 at an MOI of 0.1 for 1 h, washed to the removal of non-endocytosed virus and each protein was added to the cells were incubated for 72 h (A). Viral replication was measured by using TCID_50_ method assay (B) and western blotting assays (C). PBS was used as untreated infected control. Mean values of three independent experiments each measured in triplicate including the standard deviation are shown. Statistically significant differences between each compound and PBS control are shown. Significant differences from control denoted by **** (*p* ≤ 0.0001).

The effect of the proteins on EVA71 post-entry steps was also investigated. To perform this, Vero cells were infected with EVA71 for 1 h, washed with PBS to remove unbound virus particles, and replaced with compound-containing media (Fig. 5A). CM10 and CM14 inhibited 99 % and 100 % of viral replication, respectively (Fig. 5B). Similarly, Crotamin and PLA2_CB_ reduced EVA71 replication by 97 % and 89 %, respectively (Fig. 5B). Effects on EVA71 replication were also evaluated by detecting the EVA71 VP0 protein by western blot, confirming that replication was also reduced in the presence of CM10, CM14, Crotamin, and PLA2_CB_ (Fig. 5C). The results of the time of drug-addition assays are summarised in Table 2.

### 3.4 CM10, CM14 and Crotamin inhibit high titres of EVA71

**Fig. 6:**
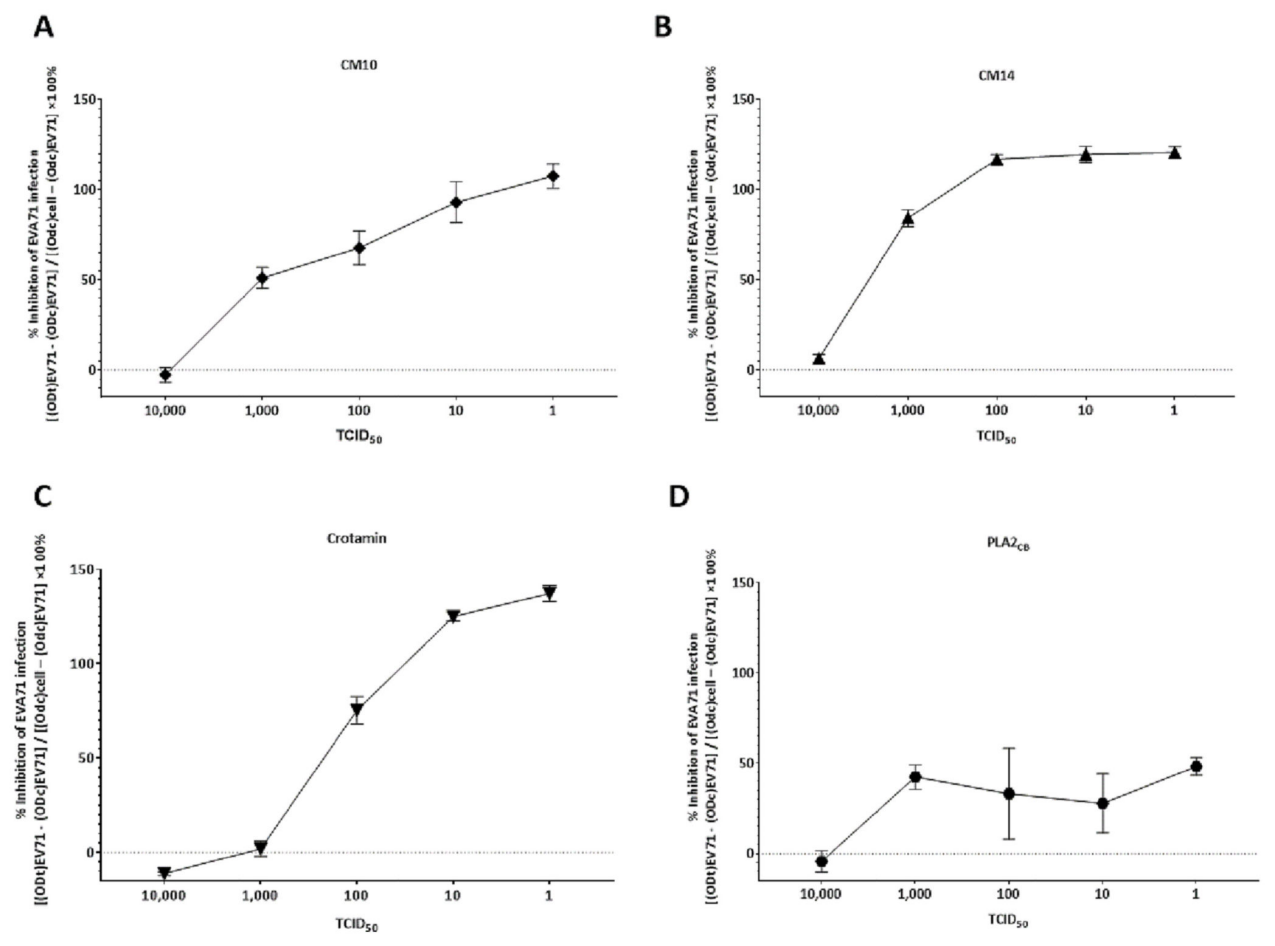
Effect of CM10, CM14, Crotamin, and PLA2_CB_ at increasing EVA71 TCID_50_ challenge doses. Vero cells were infected with 1, 10, 100, 1000, and 10,000 TCID_50_ of EVA71 in the presence of CM10, CM14, and Crotamin at 50 μg/mL or PLA2CB at 5 μg/mL. PBS was used as untreated control. After 48 h, supernatant was removed and MTT solution was added to cells. The MTT solution was removed, replaced by the same volume of DMSO, and read at a wavelength of 490 nm on the GloMax plate reader (PROMEGA). The results were then transformed into percentages, and the inhibition of EVA71 replication in cells by each protein was calculated using the following formula [(ODt)EVA71 − (ODc)EVA71] / [(Odc)cell − (Odc)EVA71] × 100 (%), where (ODt)EVA71 is the measured optical density of treatment on EVA71 infected cells; (ODc)EVA71 is the optical density measured for untreated control EVA71 infected cells; and (Odc)cell is the measured optical density of uninfected control cells [46]. Antiviral activity was presented as % inhibition of EVA71 replication. Mean values ± SD of two independent experiments each measured in triplicate are shown.

To further describe the effect of the proteins on the EVA71 replicative cycle, we aimed to analyze the capacity of each compound in inhibiting increasing concentrations of EVA71 [48,49]. To this, cells were infected with virus at 1, 10, 100, 1000, and 10,000 TCID_50_ in the presence of CM10, CM14, and Crotamin at 50 μg/mL or PLA2_CB_ at 5 μg/ mL. The results showed that none of the proteins were able to inhibit EVA71 at 10,000 TCID_50_ (Fig. 6A−D). However, CM14 inhibited EVA71 infection at 1000 TCID_50_ by 84.2 % and was able to completely impair viral replication at 100 TCID_50_ or lower titres (Fig. 6B). Alternatively, CM10 inhibited 51.1 % of the infection at 1000 TCID_50_, and 67.8 % or 93 % at 100 TCID_50_, or 10 TCID_50_, respectively (Fig. 6A). Crotamin inhibited EVA71 infection at 100 TCID_50_ (75.3 %) but not at higher doses (Fig. 6C). Lastly, PLA2_CB_ was not able to completely inhibit EVA71 infection, however, presented a similar inhibition (≈35−45 %) of infection up to 1000 TCID_50_ (Fig. 6D).

## 4 Discussion

Currently, there is no specific antiviral therapy to treat EVA71 infection, a virus associated with HFMD [50]. It is considered an endemic virus in Pacific Asia, where there are concerns about the potential of dissemination in highly populated areas [51], and it poses a threat to public health due to the viral neural tropism [52]. Therefore, the search for active compounds against EVA71 is essential.

Proteins isolated from snake venoms demonstrated antiviral activity against viruses from different families such as *Flaviviridae* [32], *Para-myxoviridae* [53], *Retroviridae* [54], *Bunyaviridae* [53], and *Togaviridae* [33,55]. However, to the best of our knowledge this is the first description of proteins isolated from snake venoms possessing antiviral activity against the *Picornaviridae* family.

The protein PLA2_CB_ investigated in this study was isolated from *C. durissus terrificus* and previously demonstrated antiviral activity against HCV [56] and Chikungunya virus (CHIKV) [55], RNA enveloped viruses belonging to the *Flaviviridae* and *Togaviridae* families, respectively. Against HCV and CHIKV, PLA2_CB_ was able to protect host cells from virus infection, reduced virus replication, and presented virucidal activity, differing from what was presented here against EVA71. In addition, a PLA2 isolated from *Vipera nikolskii* exhibited potent antiviral effect against SARS-CoV-2, presenting low cytotoxicity and decreasing virus titres by acting on virion lipidic membrane [57]. Muller and co-workers demonstrated that PLA2_CB_ was not able to inhibit the non-enveloped virus Coxsackie B5 virus (also from *Picornaviridae* family) [33,37], which corroborates with our results, and since PLA2_CB_ is a phospholipase with activity relying on membranes, we suggest that the modest SI, as well as the lack of virucidal and/or protective activity, could be related to the absence of an envelope on EVA71. Interestingly, the PLA2_CB_ effect was observed in the post entry steps of EVA71 replication. Considering that the EVA71 proteins 2B, 2C, and 3A play roles in the formation of a lipid environment for EVA71 replication, termed membranous replication organelles [58], which are essential for virus genome replication, one possible mechanism of action of PLA2_CB_ on EVA71 replication could be the disruption of these replicative structures. However, this hypothesis needs to be further investigated.

In addition, the results presented herein showed for the first time the antiviral activity of CM10 and CM14 (both from *B. moojeni*), and Crotamin (*C. durissus terrificus*) against EVA71. Crotamin is a low molecular weight cationic polypeptide [59] that is able to penetrate cell membranes [60], and was described to possess antimicrobial [61] and antifungal activities [62]. Muller and colleagues investigated the activity of Crotamin against both DENV and YFV both enveloped viruses that perturb lipid metabolism, however, this molecule did not show any significant effect against these viruses [37]. What is more interesting is that Crotamin strongly inhibited EVA71, with activity in several steps of viral replication. Crotamin is capable of inhibiting voltage-gated potassium channels, resulting in depolarization of the cell membrane, and inducing the entry of Ca^2+^ [63] and activating Ca^2+^ dependent cell signalling cascades. This molecule was also reported to be able to interact with lipid bilayers and penetrate different cell lines [64]. In addition, Crotamin was shown to disrupt endosomes and lysosomal vesicles in mammalian cells [65,66], and since EVA71 replication is dependent on the lipid environment, as well as vesicle trafficking in the cytoplasm [58], it is possible that this protein is impairing EVA71 replication by interfering with cytoplasmic membrane trafficking.

What is more, both CM10 and CM14 proteins inhibited EVA71 replication and impaired viral infection through a virucidal activity. In addition, CM14 also protected cells against EVA71 infection, being active against all stages of virus replication investigated in this study. CM10 is a glycoprotein that is also known as a moojase, possessing serine protease activity [40]. In the literature, CM10 (moojase) has not been reported to possess therapeutic potential against infectious diseases, being characterized only as a thrombin-like protein, and resulting in interactions with the coagulation cascades in mammals [40]. However, CM14 is a Lys49 phospholipase A2 homologue, also known as MjTx-II, and does not possess catalytic activity [67]. This protein exhibited antimicrobial activity against *Escherichia coli* and *Candida albicans*, as well as antiparasitic activity against *Schistosoma mansoni* and *Leishmania* spp. [68]. The mechanism of action of CM14 was associated with its ability to destabilize membranes [69,70], and in this context, we note that EVA71 hijacks the autophagy process during infection [71]. It is possible therefore that the antiviral activity of CM14 against EVA71 replication was due to an effect on cytoplasmic membranes. Even though no antiviral activity was previously reported for these two proteins from *B. moojeni*, the capacity of CM14 to interfere with several infectious pathogens demonstrates the potential of these molecules to be further exploited for antiviral development against HFMD.

Although EVA71 lacks an envelope, the virus presents a depression in the capsid termed the canyon, an important region where cellular receptors bind to the virus particle [72]. The canyon harbours a hydrophobic lipid pocket factor that is involved in regulating particle stability [73,74]. The release of a lipid pocket factor from the capsid is required for the conformational changes, which ultimately lead to viral infectivity. Based on the data presented here, CM10, CM14, and Crotamin demonstrated a significant virucidal activity and we suggest that these proteins could be acting by: 1) stabilising the particles and preventing uncoating (thus infection); 2) destabilising the particles and prematurely inducing uncoating; or 3) interacting at places on the capsid required for cell interaction or receptor engagement, thus preventing receptor binding. However, more studies are needed for a better understanding of how these compounds are acting on EVA71.

Altogether, the results demonstrated that all the evaluated proteins isolated from snake venom inhibited the EVA71 replicative cycle. Most interestingly, CM14 and Crotamin were able to significantly inhibit all stages of virus infection here investigated, with high SI. This study is the first report of proteins isolated from Brazilian snake venoms with antiviral activity against EVA71. In addition, the molecules presented here emphasize the employment of natural sources to identify antiviral candidates, as well as identify scaffold molecules for drug design.

## Supplementary Material

Supplementary data to this article can be found online at https://doi.org/10.1016/j.ijbiomac.2023.124519.

Figure S1

## Figures and Tables

**Table 1:** CC_50_, EC_50_, and Selective index of each protein.

Protein	Venom Origin	CC_50_ (μg/mL)	EC_50_(μg/mL)	SI
CM10	*B. moojeni*	427.6	6.3	67.4
CM14	*B. moojeni*	>1000	5.9	>170.8
Crotamin	*C. durissus terrificus*	>1000	8.3	>120.4
PLA2_CB_	*C. durissus terrificus*	52.3	4.2	12.5

**Table 2:** Activity of proteins isolated from snake venoms against EVA71.

Protein	Non-toxic concentration	Cell viability (%)	Protective activity (%)	Virucidal activity (%)	Inhibition of replication (%)
CM10	50 μg/mL	104	76	98	100
CM14	50 μg/mL	107	−	88	99
Crotamin	50 μg/mL	93	62	99	97
PLA2_CB_	5 μg/mL	97	−	−	89
