# Supplementary figures and images for: Effect of proteins isolated from Brazilian snakes on enterovirus A71 replication cycle: An approach against hand, foot and mouth disease
Source: Int J Biol Macromol. Author manuscript; Available in PMC 2024 Mar 4. (PMC7615699; doi:10.1016/j.ijbiomac.2023.124519)

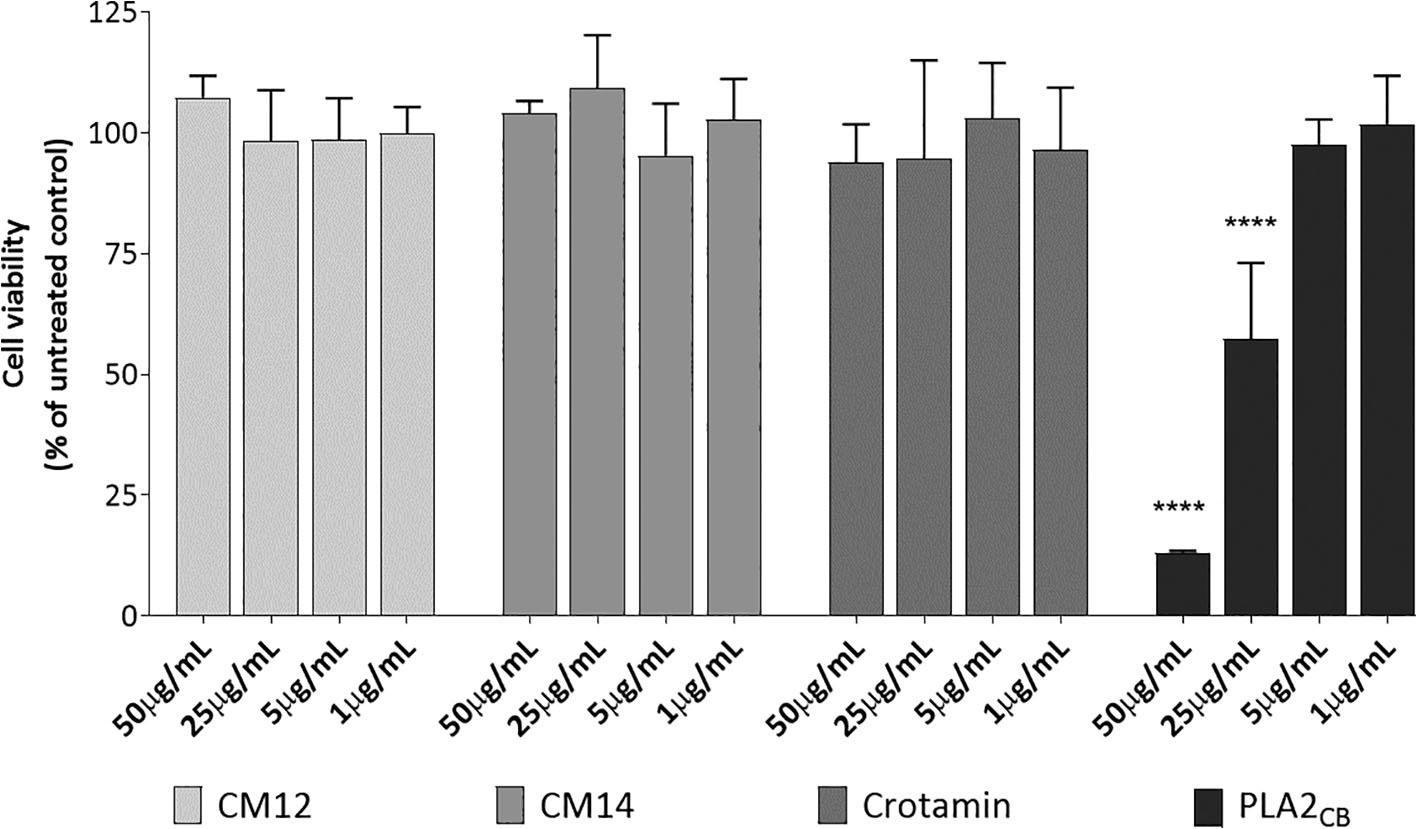

Supplement: Figure S1 [file EMS194312-supplement-Figure_S1.jpg]
